# Relationship of Distance to PCI Center With In-Hospital Mortality After STEMI: Insights From a Rural Regional STEMI Network

**DOI:** 10.1016/j.jscai.2026.104271

**Published:** 2026-02-12

**Authors:** Raihan Kabir, Jennifer Liao, Andres Cordova Sanchez, Yiwei Li, Cathy S. Ross, David W. Butzel, Ansar Hassan, James M. Flynn, Prospero Gogo, James T. DeVries, Harold L. Dauerman, Tanush Gupta

**Affiliations:** a Division of Cardiology, University of Vermont Medical Center, Burlington, Vermont; b Department of Medicine, University of Vermont Medical Center, Burlington, Vermont; c Division of Cardiology, Dartmouth Hitchcock Medical Center, Lebanon, New Hampshire; d Division of Cardiology, Maine Medical Center, Portland, Maine; e Division of Cardiac Surgery, Maine Medical Center, Portland, Maine; f Division of Cardiology, Catholic Medical Center, Manchester, New Hampshire

**Keywords:** fibrinolysis, pharmacoinvasive percutaneous coronary intervention, primary percutaneous coronary intervention, ST-elevation myocardial infarction, systems of care

## Abstract

**Background:**

Patients who present with ST-elevation myocardial infarction (STEMI) to a hospital without primary percutaneous coronary intervention (PPCI) capability may be transferred for PPCI or receive fibrinolytic therapy, depending on whether the first medical contact to balloon time of ≤120 minutes can be achieved. We hypothesized that travel distance to the percutaneous coronary intervention (PCI) center might impact STEMI outcomes in a rural STEMI system of care.

**Methods:**

We retrospectively analyzed 6225 consecutive STEMI patients from the Northern New England Cardiovascular Disease Study Group registry from 2018 to 2023. Residential distance to care was calculated by road travel distance between ZIP Code centroids and grouped into quartiles. Patients were categorized by treatment strategy: initial presentation to a PCI center, transfer for PPCI, or pharmacoinvasive PCI. The primary outcome was in-hospital mortality. Multivariable logistic regression evaluated the association between residential distance and treatment strategy with in-hospital mortality.

**Results:**

The median residential distance to PCI center for STEMI patients in Northern New England was 38.2 miles (IQR, 47.1). Of the overall cohort, 44.4% (median travel distance: 17.2 miles) had index presentation to a PCI hospital, 23.8% (median travel distance: 40.1 miles) underwent interhospital transfer for primary PCI, and 31.9% (median travel distance: 66.5 miles) were transferred for pharmacoinvasive PCI after initial lytic therapy. There was no association between distance to PCI hospital and adjusted in-hospital mortality after STEMI, irrespective of the treatment group.

**Conclusions:**

In Northern New England with contemporary regional STEMI referral networks, distance to PCI hospital did not impact STEMI mortality despite substantial travel distances for many patients.

## Introduction

Timely reperfusion is an important modifiable determinant of survival after ST-segment elevation myocardial infarction (STEMI).^1^ The 2025 American College of Cardiology/American Heart Association guidelines for the management of acute coronary syndromes recommend primary percutaneous coronary intervention (PPCI) for STEMI with a goal of the first medical contact (FMC)-to-device time of ≤90 minutes.^1^ When FMC-to-device time of ≤90 minutes is not feasible, transfer for PPCI is recommended if FMC-to-balloon time of ≤120 minutes can be achieved.^1^ Fibrinolysis followed by transfer to percutaneous coronary intervention (PCI) center (pharmacoinvasive PCI) is recommended when PPCI is not feasible within 120 minutes of FMC.^1^ The excess mortality hazard associated with reperfusion delays is well-documented across system intervals: longer total ischemic time (that includes symptom onset to FMC time, prehospital delays, and door-to-reperfusion time) correlates with higher short- and long-term mortality after STEMI.^2^, ^3^, ^4^, ^5^

Geography is a persistent upstream constraint on timely reperfusion. Although many U.S. residents live within a reasonable drive of a PPCI hospital, substantial populations—disproportionately in rural regions—remain at risk of delayed access. Even when transport is possible, rural emergency medical system patients frequently miss guideline FMC-to-PCI targets.^6^ Access constraints contribute to enduring the rural-urban gaps in cardiovascular care and outcomes, including higher mortality for acute myocardial infarction (AMI) at rural hospitals despite decades of quality initiatives.^7^ Over the last 3 decades, regionalized STEMI systems of care have been established to mitigate geography-driven delay through prehospital electrocardiogram triage, direct transport to PCI centers, standardized interhospital transfer protocols, and routine use of pharmacoinvasive therapy when timely PPCI is not feasible.^8^^,^^9^ Such systems consistently shorten reperfusion times and have been associated with improvements in in-hospital outcomes at scale.^8^^,^^9^

Prior studies examining the impact of distance to PPCI center on STEMI outcomes have shown conflicting results, with some showing a correlation between distance and mortality and others showing no impact of distance on mortality.^10^, ^11^, ^12^, ^13^ Therefore, a key question remains for contemporary regional rural STEMI networks in the United States: does residential distance to a PCI center independently influence in-hospital mortality once patients reach the invasive pathway, or have regional systems of STEMI care effectively neutralized geography? Furthermore, beyond distance, the impact of treatment strategy—direct PPCI, interhospital transfer for PPCI, or pharmacoinvasive transfer PCI—on STEMI mortality needs to be reexamined. Accordingly, we examined the impact of residential distance to PCI care for STEMI patients in the current era of delineated STEMI treatment networks in Northern New England, a region with a large rural population and a relatively small number of PCI-capable hospitals across a multistate area.

## Methods

Data were obtained from the Northern New England Cardiovascular Disease Study Group (NNECDSG) PCI registry to identify patients undergoing PCI for STEMI from 2018 to 2023. The NNECDSG is a voluntary, regional consortium of 8 medical centers in Vermont, Maine, and New Hampshire, aimed to study cardiovascular care outcomes and inform quality improvement. Data collected include demographics, comorbidities, cardiac history, presence of cardiogenic shock or cardiac arrest, PCI indication, priority, coronary anatomy, procedural characteristics, and in-hospital outcomes. Data collection is designated as a Quality Improvement Registry, and therefore, patient consent for this study was not required; patients sign consent at the time of procedure, including them in the registry and all future analyses. Data collection is validated every 2 years by comparison to administrative records.

The primary objective of this study was to assess the association between residential distance to PCI centers and in-hospital mortality. Residential distance is defined as the one–way road travel distance in miles from the geographic center of the patient’s residential ZIP Code to the center of the PCI–performing hospital ZIP Code, calculated using geographic information system software and categorized into quartiles: <12.37 miles, 12.37 to 32 miles, 32 to 60 miles, and >60 miles. In-hospital mortality is defined as death from any cause occurring during the index hospitalization. Our secondary objective was to examine the association of treatment strategy with STEMI in-hospital mortality. Treatment strategy is stratified into 3 categories: patients who either presented or were transferred directly to a PCI-capable center (initial presentation to PPCI center), patients who presented to a non-PCI hospital and then transferred for PPCI without receiving fibrinolytic therapy (transfer for PPCI), and patients who received fibrinolytic therapy at the initial hospital and then subsequently transferred for PCI (pharmacoinvasive PCI). We also studied temporal trends in reperfusion strategy for STEMI in Northern New England during the study period.

The analysis of variance or Kruskal-Wallis tests were used to compare continuous variables based on distribution, and the χ^2^ test was used to compare categorical data. Multivariable logistic regression analysis was used to evaluate the association between residential distance and treatment strategy with in-hospital mortality. The regression models adjusted for demographics, baseline comorbidities, coronary anatomy, left ventricular ejection fraction (LVEF), cardiogenic shock, and cardiac arrest. Temporal trends were evaluated using simple linear regression such that the slope represented the average annual change in proportion. All analyses were performed using R version 4.2.2 (The R Foundation), STATA version 17.0 (StataCorp), and Prism version 10.6 (GraphPad Software). A 2-tailed *P* value <.05 was considered statistically significant.

## Results

### Baseline characteristics by residential distance to PCI center

Patient demographics and comorbidities were largely similar across distance quartiles. The mean age did not significantly differ between groups (*P* = .84). There was a greater proportion of male sex with increasing distance: 70.9% in Q1 vs 75.4% in Q4 (*P* = .015). The prevalence of major cardiovascular comorbidities, including diabetes (25.3%), hypertension (65.7%), and dyslipidemia (60.4%), did not vary significantly among distance quartiles. Similarly, the rates of chronic kidney disease, chronic obstructive pulmonary disease, prior myocardial infarction, prior PCI, prior coronary artery bypass grafting, peripheral artery disease, and prior stroke were similar across quartiles. Heart failure was slightly more common among patients residing 32-60 miles from a PCI center (12.4%) compared with those living closer or farther away (Table 1).

**Table 1:** Clinical and procedural characteristics of STEMI patients by distance from PCI center

	Total (N = 6225)	Quartile 1 (<12.37 miles) (n = 1211)	Quartile 2 (12.37-32 miles) (n = 1426)	Quartile 3 (32-60 miles) (n = 1809)	Quartile 4 (>60 miles) (n = 1779)	*P* value
Age, y	63.6 ± 12.2	63.7 ± 12.4	63.4 ± 12.3	63.7 ± 12.2	63.5 ± 11.8	.84
Sex						.015
Men	4534 (72.8)	858 (70.9)	1043 (73.1)	1291 (71.4)	1342 (75.4)	
Women	1691 (27.2)	353 (29.1)	383 (26.9)	518 (28.6)	437 (24.6)	
Race—White	6060 (97.3)	1169 (94.5)	1390 (97.5)	1759 (97.2)	1742 (97.9)	.17
Comorbidities						
Diabetes	1578 (25.3)	298 (24.6)	362 (25.4)	466 (25.8)	452 (25.4)	.92
Hypertension	4087 (65.7)	811 (67.0)	931 (65.3)	1157 (64.0)	1188 (66.8)	.24
Dyslipidemia	3,760 (60.4)	725 (59.9)	858 (60.2)	1098 (60.8)	1079 (60.7)	.95
Current smoker	2090 (33.6)	379 (31.3)	473 (33.2)	627 (34.7)	611 (34.3)	.23
CKD	174 (2.8)	27 (2.2)	43 (3.0)	62 (3.4)	42 (2.4)	.13
COPD	656 (10.5)	135 (11.1)	134 (9.4)	204 (11.3)	183 (10.3)	.30
Heart failure	655 (10.5)	108 (8.9)	146 (10.2)	225 (12.4)	176 (9.9)	.011
Prior MI	986 (15.8)	202 (16.7)	247 (17.3)	284 (15.7)	253 (14.2)	.089
Prior PCI	1,147 (18.4)	232 (19.2)	277 (19.4)	340 (18.8)	298 (16.8)	.18
Prior CABG	243 (3.9)	46 (3.8)	64 (4.5)	69 (3.8)	64 (3.6)	.61
PAD	703 (11.3)	135 (11.1)	164 (11.5)	210 (11.6)	194 (10.9)	.91
Prior stroke	352 (5.7)	62 (5.1)	90 (6.3)	105 (5.8)	95 (5.3)	.53
Cardiac arrest	610 (9.8)	121 (10.0)	144 (10.1)	158 (8.7)	187 (10.5)	.3
Cardiogenic shock	535 (8.6)	109 (9.0)	122 (8.6)	164 (9.1)	140 (7.9)	.58
No. of vessels diseased						.98
0	131 (2.1)	26 (2.1)	31 (2.2)	37 (2.0)	37 (2.1)	
10	3730 (59.9)	720 (59.5)	853 (59.8)	1080 (59.7)	1077 (60.5)	
20	1678 (27.0)	321 (26.5)	384 (26.9)	488 (27.0)	485 (27.3)	
30	686 (11.0)	144 (11.9)	158 (11.1)	204 (11.3)	180 (10.1)	
Left main stenosis >50%	272 (4.4)	51 (4.2)	56 (3.9)	92 (5.1)	73 (4.1)	.35
LAD stenosis ≥70%	3425 (55.0)	668 (55.2)	782 (54.8)	987 (54.6)	988 (55.5)	.95
RCA stenosis ≥70%	3630 (58.3)	698 (57.6)	835 (58.6)	1068 (59.0)	1029 (57.8)	.85
LCX stenosis ≥70%	2089 (33.6)	428 (35.3)	478 (33.5)	613 (33.9)	570 (32.0)	.30
LVEF						
LVEF, %	46.6 ± 14.1	47.9 ± 13.3	47.0 ± 15.0	46.5 ± 13.8	45.6 ± 14.0	.66
<40%	156 (2.5)	21 (1.7)	38 (2.7)	50 (2.8)	47 (2.6)	.29
Treatment strategy						<.001
Initial presentation for PPCI	2763 (44.4)	1045 (86.3)	910 (63.8)	516 (28.5)	292 (16.4)	
Transfer for PPCI	1479 (23.8)	145 (12.0)	380 (26.6)	627 (34.7)	327 (18.4)	
Pharmacoinvasive PCI	1983 (31.9)	21 (1.7)	136 (9.5)	666 (36.8)	1160 (65.2)	
Arterial access site						<.001
Femoral	1648 (26.5)	349 (28.8)	431 (30.2)	473 (26.1)	395 (22.2)	
Brachial	10 (0.2)	2 (0.2)	2 (0.1)	4 (0.2)	2 (0.1)	
Radial	4549 (73.1)	857 (70.8)	991 (69.5)	1323 (73.1)	1378 (77.5)	
Other	18 (0.3)	3 (0.2)	2 (0.1)	9 (0.5)	4 (0.2)	
PPCI vessel^a^						
LM	49 (0.8)	12 (1.0)	6 (0.4)	16 (0.9)	15 (0.8)	.34
LAD	2265 (36.4)	431 (35.6)	530 (37.2)	641 (35.4)	663 (37.3)	.57
Diagonal	190 (3.1)	36 (3.0)	41 (2.9)	50 (2.8)	63 (3.5)	.55
LCX	850 (13.7)	185 (15.3)	184 (12.9)	252 (13.9)	229 (12.9)	.22
Ramus	61 (1.0)	9 (0.7)	15 (1.1)	15 (0.8)	22 (1.2)	.49
RCA	2856 (45.9)	546 (45.1)	655 (45.9)	852 (47.1)	803 (45.1)	.62
Preintervention TIMI flow^a^						<.001
TIMI 0	2928 (47)	740 (61.3)	817 (57.3)	793 (43.8)	578 (32.5)	
TIMI 1	463 (7.5)	96 (7.9)	103 (7.2)	132 (7.3)	132 (7.4)	
TIMI 2	884 (14.2)	146 (12.1)	178 (12.5)	272 (15.1)	288 (16.2)	
TIMI 3	1939 (31.2)	226 (18.7)	326 (22.9)	607 (33.6)	780 (43.8)	
Missing	11 (0.1)	3 (0.2)	2 (0.1)	5 (0.3)	1 (0.1)	
Postintervention TIMI flow^a^						.081
TIMI 0	54 (0.9)	10 (0.8)	13 (0.9)	17 (0.9)	14 (0.8)	
TIMI 1	38 (0.6)	5 (0.4)	7 (0.5)	21 (1.2)	5 (0.3)	
TIMI 2	153 (2.4)	34 (2.8)	30 (2.1)	47 (2.6)	42 (2.4)	
TIMI 3	5881 (94.5)	1142 (94.3)	1353 (94.9)	1692 (93.5)	1694 (95.2)	
Missing	99 (1.6)	20 (1.7)	23 (1.6)	32 (1.8)	24 (1.3)	
In-stent restenosis^a^	297 (4.8)	58 (4.8)	65 (4.6)	84 (4.6)	90 (5.1)	.93
In-stent thrombosis^a^	207 (3.3)	52 (4.3)	56 (3.9)	58 (3.2)	41 (2.3)	.012
Lesion in graft^a^	122 (2.0)	22 (1.8)	32 (2.2)	40 (2.2)	28 (1.6)	.44
Type of CABG graft^a^						.43
LIMA	11 (0.17)	3 (0.2)	5 (0.3)	1 (0.1)	2 (0.11)	
SVG	109 (1.8)	19 (1.6)	27 (1.9)	38 (1.9)	25 (1.41)	
Other artery	2 (0.03)	0 (0.0)	0 (0.0)	1 (0.1)	1 (0.06)	
No lesion in graft	6103 (98)	1189 (98.2)	1394 (97.8)	1769 (97.8)	1751 (98.42)	
Lesion complexity^a^						<.001
Non-high/Non-complex	1097 (17.6)	208 (17.2)	208 (14.5)	318 (17.6)	363 (20.4)	
High/complex	5112 (82.1)	1000 (82.6)	1213 (85.1)	1484 (82)	1415 (79.5)	
Missing	16 (0.3)	3 (0.2)	5 (0.4)	7 (0.4)	1 (0.1)	
Severe calcification^a^	285 (4.7)	58 (4.9)	74 (5.4)	77 (4.4)	76 (4.4)	.51
Bifurcation lesion^a^	559 (9.0)	109 (9.0)	128 (9.0)	166 (9.2)	156 (8.8)	.98

Values are mean ± SD or n (%).

CABG, coronary artery bypass grafting; CKD, chronic kidney disease; LAD, left anterior descending; LCX, circumflex; LIMA, left infernal mammary artery; MI, myocardial infarction; PAD, peripheral artery disease; PCI, percutaneous coronary intervention; PPCI, primary percutaneous coronary intervention; RCA, right coronary artery; STEMI, ST-segment elevation myocardial infarction; SVG, saphenous vein graft; TIMI, Thrombolysis in Myocardial Infarction.

a In culprit lesion only.

The proportion of cardiogenic shock or cardiac arrest complicating STEMI was similar across distance groups. There were no significant differences in coronary anatomy or left ventricular function across groups (Table 1).

As expected, travel distance was associated with differences in reperfusion strategy. Patients living closer to PCI centers were more likely to present directly to a PPCI center (86.3%, 63.8%, 28.5%, and 16.4%, respectively, in quartiles 1-4, respectively, *P* < .001). Conversely, transfer for PPCI or pharmacoinvasive PCI was more common with increasing travel distance (Table 1). In distance quartile 4 (travel distance >60 miles), approximately 1/3 of patients received pharmacoinvasive PCI.

### Baseline characteristics by treatment strategy

Of the overall cohort of STEMI patients, 2763 (44.4%) presented directly to the PPCI center, 1479 (23.8%) underwent interhospital transfer for PPCI, and 1983 (32.6%) underwent pharmacoinvasive PCI. The patients in the transfer after fibrinolytics group were younger than those in other groups (mean [± SD] 62.5 [± 11.8] vs 64.0 [± 12.3] and 64.1 [± 12.4] years, respectively; *P* < .001), whereas sex distribution was similar across all groups (approximately 73% men, *P* = .41) (Table 2).

**Table 2:** Clinical and procedural characteristics of STEMI patients by treatment strategy.

	Total STEMI (N = 6225)	Initial presentation to PPCI center (n = 2763)	Transfer for PPCI (n = 1479)	Pharmacoinvasive PCI (n = 1983)	P value
Age, y	63.6 ± 12.2	64.0 ± 12.3	64.1 ± 12.4	62.5 ± 11.8	<.001
Sex					.41
Men	4534 (72.8)	2001 (72.4)	1067 (72.1)	1466 (73.9)	
Women	1691 (27.2)	762 (27.6)	412 (27.9)	517 (26.1)	
Race—White	6060 (97.3)	2689 (97.3)	1444 (97.6)	1927 (97.2)	.71
Comorbidities					
Diabetes	1578 (25.3)	715 (25.9)	360 (24.3)	503 (25.4)	.55
Hypertension	4087 (65.7)	1831 (66.3)	956 (64.7)	1300 (65.6)	.58
Dyslipidemia	3760 (60.4)	1693 (61.3)	876 (59.3)	1191 (60.1)	.40
Current smoker	2090 (33.6)	867 (31.4)	483 (32.7)	740 (37.3)	<.001
CKD	174 (2.8)	86 (3.1)	41 (2.8)	47 (2.4)	.31
COPD	656 (10.5)	284 (10.3)	154 (10.4)	218 (11.0)	.72
Heart failure	655 (10.5)	294 (10.6)	194 (13.1)	167 (8.4)	<.001
Prior MI	986 (15.8)	488 (17.7)	218 (14.7)	280 (14.1)	.002
Prior PCI	1147 (18.4)	569 (20.6)	261 (17.6)	317 (16.0)	<.001
Prior CABG	243 (3.9)	113 (4.1)	65 (4.4)	65 (3.3)	.19
PAD	703 (11.3)	329 (11.9)	184 (12.4)	190 (9.6)	.012
Prior stroke	352 (5.7)	170 (6.2)	89 (6.0)	93 (4.7)	.078
Cardiac arrest	610 (9.8)	125 (4.5)	280 (18.9)	205 (10.3)	<.001
Cardiogenic shock	535 (8.6)	213 (7.7)	188 (12.7)	134 (6.8)	<.001
No. of vessels diseased					<.001
0	131 (2.1)	47 (1.7)	34 (2.3)	50 (2.5)	
1	3730 (59.9)	1621 (58.7)	881 (59.6)	1228 (61.9)	
2	1678 (27.0)	750 (27.1)	389 (26.3)	539 (27.2)	
3	686 (11.0)	345 (12.5)	175 (11.8)	166 (8.4)	
Left main stenosis >50%	272 (4.4)	126 (4.6)	83 (5.6)	63 (3.2)	.002
LCX stenosis ≥70%	2089 (33.6)	986 (35.7)	512 (34.6)	591 (29.8)	<.001
LAD stenosis ≥70%	3425 (55.0)	1539 (55.7)	841 (56.9)	1045 (52.7)	.032
RCA stenosis ≥70%	3630 (58.3)	1631 (59.0)	831 (56.2)	1168 (58.9)	.16
LVEF, %	46.6 ± 14.1	45.8 ± 13.7	47.2 ± 14.3	47.2 ± 14.4	.51
<40	156 (2.5)	69 (2.5)	44 (3.0)	43 (2.2)	.32
Distance to PCI hospital (miles)					<.001
<12.37	1211 (19.5)	1045 (37.8)	145 (9.8)	21 (1.1)	
12.37-32	1426 (22.9)	910 (32.9)	380 (25.7)	136 (6.9)	
32-60	1809 (29.1)	516 (18.7)	627 (42.4)	666 (33.6)	
>60	1779 (28.6)	292 (10.6)	327 (22.1)	1160 (58.5)	
Arterial access site					<.001
Femoral	1648 (26.5)	778 (28.2)	471 (31.8)	399 (20.1)	
Brachial	10 (0.2)	6 (0.2)	0 (0.0)	4 (0.2)	
Radial	4549 (73.1)	1971 (71.3)	1003 (67.8)	1575 (79.4)	
Other	18 (0.3)	8 (0.3)	5 (0.3)	5 (0.3)	
PCI vessel^a^					
LM	49 (0.8)	26 (0.9)	11 (0.7)	12 (0.6)	.42
LAD	2265 (36.4)	981 (35.5)	583 (39.4)	701 (35.4)	.021
Ramus	61 (1.0)	29 (1.0)	13 (0.9)	19 (1.0)	.86
Diagonal	190 (3.1)	67 (2.4)	53 (3.6)	70 (3.5)	.037
LCX	850 (13.7)	385 (13.9)	217 (14.7)	248 (12.5)	.16
RCA	2856 (45.9)	1288 (46.6)	616 (41.6)	952 (48.0)	<.001
Preintervention TIMI flow^a^					<.001
TIMI 0	2928 (47)	1663 (60.2)	798 (53.9)	467 (23.5)	
TIMI 1	463 (7.4)	209 (7.6)	124 (8.4)	130 (6.6)	
TIMI 2	884 (14.2)	336 (12.2)	195 (13.2)	353 (17.8)	
TIMI 3	1939 (31.2)	552 (19.9)	359 (24.3)	1028 (51.8)	
Missing	11 (0.2)	3 (0.1)	3 (0.2)	5 (0.3)	
Postintervention TIMI flow^a^					.29
TIMI 0	54 (0.8)	21 (0.8)	12 (0.8)	21 (1)	
TIMI 1	38 (0.6)	19 (0.7)	10 (0.7)	9 (0.5)	
TIMI 2	153 (2.5)	76 (2.7)	38 (2.5)	39 (1.9)	
TIMI 3	5881 (94.5)	2593 (93.8)	1397 (94.5)	1891 (95.4)	
Missing	99 (1.6)	54 (2)	22 (1.5)	23 (1.2)	
Door-to-device time, min^b^	82 (52-140)	60 (43-83)	137 (104-213)	NA	<.001
In-stent restenosis^a^	297 (4.8)	140 (5.1)	61 (4.1)	96 (4.8)	.38
In-stent thrombosis^a^	207 (3.3)	119 (4.3)	46 (3.1)	42 (2.1)	<.001
Lesion in graft^a^	122 (2.0)	57 (2.1)	33 (2.2)	32 (1.6)	.38
Type of CABG graft^a^					.16
LIMA	11 (0.17)	9 (0.33)	1 (0.1)	1 (0.1)	
SVG	109 (1.8)	47 (1.7)	32 (2.1)	30 (1.4)	
Other artery	2 (0.03)	1 (0.04)	0 (0.0)	1 (0.1)	
No lesion in graft	6103 (98)	2706 (97.93)	1446 (97.8)	1951 (98.4)	
Lesion complexity^a^					<.001
Non-high/non-complex	1097 (17.7)	440 (16.0)	233 (15.8)	424 (21.4)	
High/complex	5112 (82.3)	2311 (84.0)	1244 (84.2)	1557 (78.6)	
Missing	16 (0.3)	12 (0.4)	2 (0.1)	2 (0.1)	
Severe calcification^a^	285 (4.7)	144 (5.4)	67 (4.7)	74 (3.8)	.056
Bifurcation lesion^a^	559 (9.0)	240 (8.7)	150 (10.1)	169 (8.5)	.20

Values are mean ± SD, n (%), or median (IQR).

CABG, coronary artery bypass grafting; CKD, chronic kidney disease; LAD, left anterior descending; LCX, left circumflex; LIMA, left infernal mammary artery; MI, myocardial infarction; PAD, peripheral artery disease; PCI, percutaneous coronary intervention; PPCI, primary percutaneous coronary intervention; RCA, right coronary artery; STEMI, ST-segment elevation myocardial infarction; TIMI, Thrombolysis in Myocardial Infarction.

a Culprit lesion only.

b Door-to-Device time only available for patients for patients treated with primary PCI. Door time refers to presentation time at PPCI center for patients directly presenting to PPCI center and time of presentation at index hospital for patients transferred for PPCI. Presentation time at referring facility was not available for estimation of door-to-needle time for patients who received pharmacoinvasive PCI.

There were no significant differences in comorbidities such as diabetes, hypertension, dyslipidemia, chronic kidney disease, and chronic obstructive pulmonary disease among treatment groups. Patients transferred for PPCI had a higher prevalence of heart failure (13.1%) and peripheral artery disease (12.4%) compared with the pharmacoinvasive PCI group (8.4% and 9.6%, respectively; *P* < .001 and *P* = .012). Cardiac arrest (18.9%) and cardiogenic shock (12.7%) were most prevalent in patients transferred for PPCI (Table 2).

The prevalence of 3-vessel disease was slightly lower in the fibrinolytic group than in the PPCI group. Left main disease was also less frequent among the pharmacoinvasive PCI group (3.2%) compared with initial presentation to the PPCI center (4.6%) and transfer for PPCI (5.6%) groups (*P* = .002). LVEF did not differ significantly by treatment strategy (mean: 46.6% ± 14.1%, *P* = .51) nor did the proportion of patients with LVEF <40% (Table 2). Pre-PCI Thrombolysis in Myocardial Infarction (TIMI)-3 flow in the culprit vessel was more common in the pharmacoinvasive PCI group (52.0%) compared with patients presenting to the PPCI center (20.0%) or those transferred for PPCI (24.3%) (*P* < .001).

### In-hospital mortality by residential distance to PCI center

**Figure 1:**
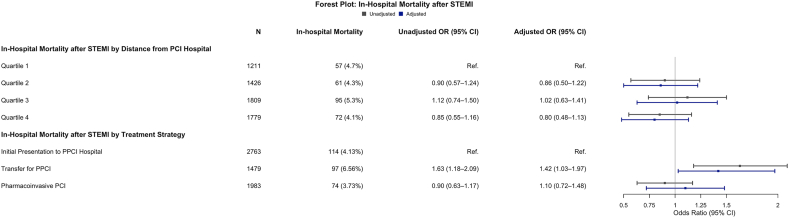
**In-hospital mortality after STEMI by distance from PCI hospital and treatment strategy.** Multivariable models adjusted for demographics, comorbidities, coronary anatomy, left ventricular ejection fraction, and presence of cardiogenic shock. PPCI, primary percutaneous coronary intervention; STEMI, ST-segment elevation myocardial infarction.

In-hospital mortality in the overall cohort was 4.6%. Crude in-hospital mortality rates were 4.7%, 4.3%, 5.3%, and 4.1%, respectively, in the distance quartile groups. Compared with patients living in distance quartile 1, in-hospital mortality was similar in distance quartiles 2 to 4 after adjusting for baseline clinical variables—including age, sex, comorbidities, presence of cardiogenic shock, LVEF, and coronary anatomy (Figure 1). These findings demonstrate that residential distance to PCI care was not associated with in-hospital mortality in this regional STEMI system.

### In-hospital mortality by treatment strategy

The highest observed in-hospital mortality occurred among patients transferred for PPCI, with a crude mortality rate of 6.6%, compared with 4.1% among patients presenting directly to the PPCI center and 3.7% among STEMI patients undergoing pharmacoinvasive PCI. After adjusting for baseline clinical characteristics and presence of cardiogenic shock or cardiac arrest, the increased mortality odds in the interhospital transfer for the PPCI group were no longer significant (adjusted odds ratio, 0.91; 95% CI, 0.64-1.29; *P* = .59) (Figure 1).

### Treatment group trends over time

**Figure 2:**
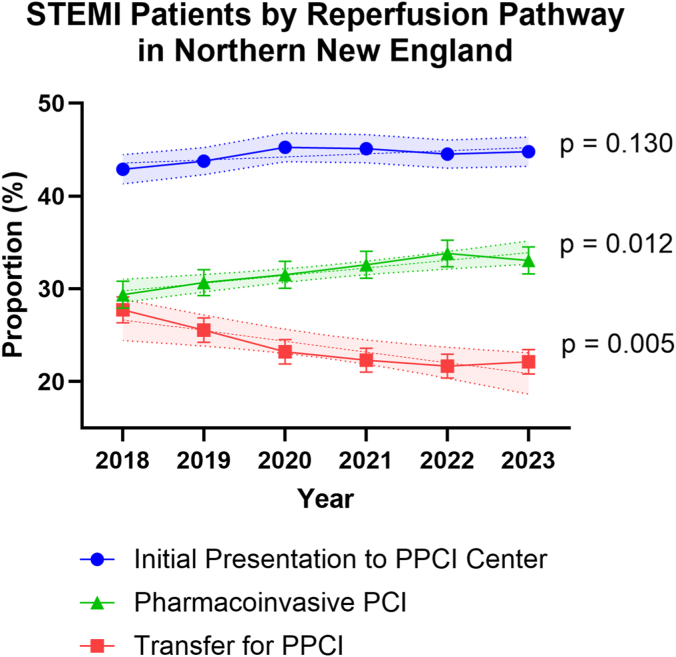
**Temporal trends in reperfusion strategy among STEMI patients in Northern New England, 2018-2023.** Points represent annual proportions of patients in each reperfusion pathway, with lines depicting linear regression fit ± 95% CI.

There were temporal changes in reperfusion strategy during the study period (Figure 2). Linear trend analysis demonstrated that the proportion of patients presenting directly to PCI centers remained stable over time (*m* = 0.33 percent/year, *P* = .13). In contrast, the proportion of patients transferred for primary PCI significantly declined (*m* = −1.16 percent/year, *P* = .012), whereas the proportion of patients transferred after fibrinolytic therapy significantly increased (*m* = 0.83 percent/year, *P* = .005).

## Discussion

In this study, we report how residential distance to PCI center influences STEMI treatment strategy and in-hospital mortality in a large, regional STEMI network serving a predominantly rural population across Northern New England. Our main findings can be summarized as follows. First, the reperfusion strategy for STEMI varies by distance to PCI center, with a greater proportion of patients receiving pharmacoinvasive PCI with increasing travel distances. Second, distance to PCI center was not independently associated with STEMI in-hospital mortality, suggesting that guideline–recommended reperfusion triage strategies with patients who live far from the PCI center initially receiving fibrinolytic therapy can mitigate treatment challenges associated with geographic remoteness (Central Illustration). Third, after risk adjustment for baseline comorbidities and presence of cardiogenic shock or cardiac arrest, STEMI patients had similar in-hospital mortality irrespective of reperfusion strategy. Fourth, during the study period, there was a decline in patients undergoing interhospital transfer for PPCI and an increase in those receiving pharmacoinvasive PCI.

**Central Illustration:**
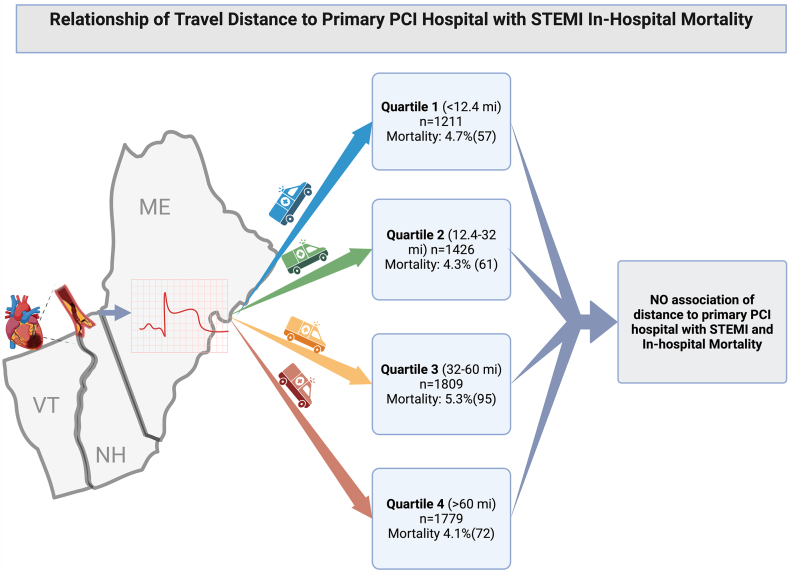
**Relationship of travel distance to primary PCI hospital with STEMI in-hospital mortality.** PCI, percutaneous cornary intervention; STEMI, ST-segment elevation myocardial infarction.

The rural nature of the STEMI population in Northern New England is reflected in the finding that the median travel distance to PCI hospital was 38 miles, and >25% of the patients had a travel distance of >60 miles. The predominant treatment strategy in the first 2 distance quartile groups was initial presentation to a PPCI hospital, whereas in patients living the farthest away, almost 2/3 received fibrinolytics as the initial reperfusion strategy. Given that the current guidelines recommend fibrinolysis in STEMI when a FMC-to-balloon time of ≤120 minutes cannot be achieved, the treatment patterns according to distance reflect adherence of our regional STEMI triage system to clinical practice guidelines.^1^

Despite geographic challenges and substantial travel distance, our study reports that in-hospital STEMI mortality does not vary with distance to the PCI hospital in our region. Prior data on distance to care and STEMI mortality have been conflicting.^10^, ^11^, ^12^, ^13^ A study from Italy showed that travel time was positively associated with 30-day mortality, and in fact, the correlation between shorter door-to-balloon time and mortality disappeared when travel times exceeded the median in that analysis.^14^ A population-based study from the Swiss National Cohort demonstrated a positive correlation between driving time to the nearest university hospital and AMI mortality.^15^ Importantly, this analysis accounted for all AMI deaths: prehospital, in-hospital, or postdischarge.^15^ Similarly, analysis of AMI deaths in Arkansas, with a large rural population, also showed that AMI mortality increased with increased drive times to the nearest PCI hospital.^16^ On the contrary, a study from the Veterans Affairs Clinical Assessment, Reporting, and Tracking Program showed no association between patient distance to PCI center and 1-year death, irrespective of PCI indication being for acute coronary syndromes or stable coronary artery disease.^12^ However, this analysis is likely confounded by the fact that veterans with long travel time to a VA PCI facility may have been triaged to a non-VA facility for PPCI in the emergent setting of STEMI.^12^ Our study suggests that in our regional STEMI system, protocolized triage and transfer protocols have neutralized the effect of residential distance on STEMI hospital mortality.

When stratified by STEMI reperfusion strategy, patients who underwent interhospital transfer for PPCI had modestly higher crude in-hospital mortality compared with patients who either presented directly to the PPCI hospital or underwent pharmacoinvasive PCI. It is well established that incremental delay from symptom onset to open artery time diminishes the benefit of PPCI.^1^^,^^17^, ^18^, ^19^ Although there have been dramatic improvements in prehospital STEMI triage and protocolization of STEMI pathways at PPCI centers, often bypassing emergency room triage, door-in-door-out times (DIDO) from the index hospital emergency room are frequently more than guideline-recommended duration of 30 minutes.^20^ In an analysis of 14,821 STEMI patients in the ACTION Registry-Get with the Guidelines, median DIDO times were 68 minutes, and only 11% of the patients had DIDO times of <30 minutes.^21^ Moreover, STEMI hospital mortality was higher in STEMI patients who achieved DIDO times of >30 minutes vs <30 minutes.^21^ Dauerman et al^22^ previously showed that of patients undergoing interhospital transfer for PPCI, one-third fail to achieve a door-to-device time of ≤120 minutes, despite a median estimated transfer distance and transfer times of 26.5 miles and <60 minutes, respectively. We did not have access to DIDO times in our data and could not account for whether higher DIDO times contributed to higher crude mortality in the PPCI transfer group. Among the 3 study groups, the proportion of STEMI patients with cardiac arrest and cardiogenic shock was highest in those who underwent interhospital transfer for PPCI. Cardiogenic shock and cardiac arrest are both relative contraindications for fibrinolytic therapy. It is likely that patients with cardiogenic shock and/or cardiac arrest complicating STEMI presenting to non-PPCI hospitals who would have otherwise qualified for initial lytic therapy based on regional triage protocols were instead transferred for PPCI without lytic therapy, increasing the risk profile of this group. After risk adjustment, there was no difference in in-hospital mortality between patients who presented directly to the PPCI hospital, underwent interhospital transfer for PPCI, or underwent pharmacoinvasive PCI. These findings emphasize the continued importance of fibrinolytic therapy for STEMI in geographically remote areas. In patients who live far from PCI center, pharmacoinvasive approach with initial lytic therapy to open infarct-related artery and prompt transfer to a PCI center is associated with similar in-hospital mortality to patients who undergo PPCI.

### Study limitations

First, although the NNECDSG registry captures robust, multicenter data across a rural region, residual confounding may persist despite multivariable adjustment. Second, there was a large proportion of missing data on time-stamp intervals (symptom-to-FMC, DIDO, and FMC-to-device). Third, distance was derived from ZIP-centroid road miles from patient’s residence, which does not account for STEMI event location and transport mode, depending on weather conditions. Finally, we could not account for prehospital or postdischarge deaths, limiting a definitive conclusion on the effect of distance–related reperfusion delays on overall STEMI mortality.

## Conclusions

In our regional STEMI system of care with guideline–recommended triage protocols, our study found that residential distance to PCI centers was not independently associated with STEMI hospital mortality in a contemporary cohort of more than 6000 STEMI patients. Similarly, risk-adjusted in-hospital mortality was similar in STEMI patients regardless of treatment strategy. Taken together, these findings underscore the significance of optimizing triage pathways in reperfusion for STEMI and reinforce the continued importance of pharmacoinvasive strategy in geographically remote settings.

## Declaration of competing interest

None of the authors have any relevant conflicts of interest to disclose.
